# Prevalence of thinness in children and adolescents in the Seychelles: comparison of two international growth references

**DOI:** 10.1186/1475-2891-10-65

**Published:** 2011-06-09

**Authors:** Pascal Bovet, Nathalie Kizirian, George Madeleine, Monika Blössner, Arnaud Chiolero

**Affiliations:** 1 Section of Noncommunicable Diseases, Ministry of Health, Victoria, Republic of Seychelles; 2 Institute of Social and Preventive Medicine (IUMSP), University Hospital Centre and University of Lausanne, rue de la Corniche 2, 1066 Epalinges, Switzerland; 3 Department of Nutrition for Health and Development, WHO, World Health Organization, Geneva, Switzerland

## Abstract

**Background:**

Thinness in children and adolescents is largely under studied, a contrast with abundant literature on under-nutrition in infants and on overweight in children and adolescents. The aim of this study is to compare the prevalence of thinness using two recently developed growth references, among children and adolescents living in the Seychelles, an economically rapidly developing country in the African region.

**Methods:**

Weight and height were measured every year in all children of 4 grades (age range: 5 to 16 years) of all schools in the Seychelles as part of a routine school-based surveillance program. In this study we used data collected in 16,672 boys and 16,668 girls examined from 1998 to 2004. Thinness was estimated according to two growth references: i) an international survey (IS), defining three grades of thinness corresponding to a BMI of 18.5, 17.0 and 16.0 kg/m^2 ^at age 18 and ii) the WHO reference, defined here as three categories of thinness (-1, -2 and -3 SD of BMI for age) with the second and third named "thinness" and "severe thinness", respectively.

**Results:**

The prevalence of thinness was 21.4%, 6.4% and 2.0% based on the three IS cut-offs and 27.7%, 6.7% and 1.2% based on the WHO cut-offs. The prevalence of thinness categories tended to decrease according to age for both sexes for the IS reference and among girls for the WHO reference.

**Conclusion:**

The prevalence of the first category of thinness was larger with the WHO cut-offs than with the IS cut-offs while the prevalence of thinness of "grade 2" and thinness of "grade 3" (IS cut-offs) was similar to the prevalence of "thinness" and "severe thinness" (WHO cut-offs), respectively.

## Background

Although the prevalence of overweight is increasing worldwide, underweight remains a major public health problem and is a leading cause of the burden of disease in low income countries [1]. Underweight is linked to growth faltering and is associated with increased morbidity and mortality [2-6]. Monitoring growth and nutritional status during infancy and childhood is therefore of primary importance. Thinness can be a marker of malnutrition although thin children are not necessarily undernourished. Thinness in school children and adolescents is largely under studied, contrasting with the vast amount of literature on infant malnutrition and a current focus on overweight in children and adolescents.

Recently, two growth references have been developed for the classification of overweight and thinness in school children and adolescents and both apply the indicator of body mass index (BMI) for age [7,8]. In 2007, Cole *et al*. [7] developed three grades of thinness, based on an international survey (hereafter referred to as "IS" cut-offs) of nationally representative samples of children aged 6 to 18 years, between 1963 and 1993. IS cut-offs were determined using the LMS method previously applied in children to assess overweight and obesity [9]. Also in 2007, the World Health Organization (WHO) released a growth reference for 5-19 years [8] based on a representative sample of children aged 1-17 years collected between 1963 and 1974 in the United States [10], merged with the WHO Child Growth Standards that were based on data collected between 1997 and 2003 in children aged 18-71 months living in favourable socioeconomic conditions in six developed and developing countries [11,12].

Few studies have compared the prevalence of thinness categories in school-aged children based on the IS and the WHO cut-offs [13-16]. In studies in Russia [16] and Brazil [13,14], the prevalence of thinness was higher when assessed with the WHO than with the lS cut-offs, while the opposite was found in Bolivia [15]. It is interesting to note that no study defining thinness using the new IS or WHO cut offs was included in a recent review of 369 studies from76 different countries that examined the nutritional status, including thinness, of school aged children [17]. This further stresses the need for studies assessing the performance of the IS and WHO cut-offs of thinness in populations.

In this paper, we compare the prevalence of thinness categories based on the IS cut-offs and the WHO cut-offs [7,8] using a large nationally representative sample of children and adolescents in the Republic of Seychelles.

## Methods and population

The Seychelles is an economically rapidly developing small island state in the African region, where a high prevalence of overweight/obesity has been documented in adults and children [18-22]. Under-nutrition is no longer considered a major public health problem in the Seychelles, in contrast to several other countries in the region.

This study used the pooled data set of the annual school-based surveillance program conducted in the Seychelles between 1998 and 2004. All children attend obligatory school up to the 10^th ^grade in the Seychelles. The majority of the population is of African descent with minorities of European, Indian, and Chinese origins. The sampling frame, measurement methods and results for overweight/obesity have been published previously [19,23].

Briefly, data were collected every year by approximately 20 school nurses in all children in four selected grades of obligatory school: kindergarten, 4^th^, 7^th ^and 10^th ^grades, in children aged [mean (± SD)] 5.4 (±0.4), 9.1 (±0.4), 12.5 (±0.4), and 15.6 (±0.5) years, respectively (~6000 children were screened every year). Eligible children consist of the entire population of the country. A written consent was sought from the parents before screening as part of the routine procedure. Virtually no parents or children declined participation. Because nurses performing the screening in schools also have regular duties in health centres, screening in schools may not always be as completed as expected. This underlies that a main reason for non participation of children to the screening program is likely due to random factors and not to students' personal characteristics.

Weight was measured to the nearest 0.1 kg with subjects dressed in light garments and without shoes using precision electronic scales (Seca 879, Seca, Hamburg, Germany). Height was measured to the nearest 0.1 cm against a wall using fixed stadiometers (Seca 870, Seca, Hamburg, Germany). The equipment was regularly checked for accuracy and school nurses were trained every year on the measurement procedures. BMI was calculated as the ratio of weight to height squared (kg/m^2^). Thinness was determined using the age and sex specific IS and the WHO cut-offs, as provided in tables in the original papers referring to these standards [7,8]. The IS cut-offs define three grades of thinness based on centiles passing at age 18 years through BMI 18.5, 17.0 and 16.0 kg/m^2^, respectively [7]. The WHO cut-offs are provided for -1, -2 and -3 standard deviations (SD) for BMI-for-age, of which <-2 SD defines "thinness" and <-3 SD "severe thinness" [8].

We generated, separately for boys and girls, third degree polynomial functions of BMI cut-offs according to age for each thinness category based on the BMI cut-off values tabulated at age intervals of 0.5 year for the IS [7] and age intervals of 1 month for WHO (http://www.who.int/growthref). Thinness (yes or no) for the different IS and WHO grades was defined for each child (i.e. at individual level) by comparing the actual age and sex specific BMI to the BMI predicted by the polynomial age and sex specific function. Analyses were performed with Stata 10 (Stata Corp LP, College Station, USA). Results are expressed as mean ± SD for continuous variables and as percentage and (95% confidence interval) for prevalence. Because results refer to the entire population of children and adolescents of the country, confidence intervals may not be informative in this situation.

## Results

From a total of 43,259 eligible children seen one or more times between 1998 and 2006, weight and height were available for 33,340 children (16,672 boys, 16,668 girls), corresponding to an overall participation rate of 77%. Because surveys took place every year in the four selected grades, and same children were expected to be seen at intervals of 3-4 years as they move through higher grades, observations among 33,340 children during 9 years corresponded to observations in 19'764 different children.

Table 1 illustrates the distribution of selected anthropometric data by sex and school grade. At any given age, boys tended to be taller than girls, particularly in the upper school grades, whereas girls tended to have higher BMI than boys. The prevalence for overweight including obesity displayed in Table 1 was calculated using the International Obesity Taskforce (IOTF) cut-offs [9]. The prevalence was highest for the 7^th ^grade with 13% and 19% for boys and girls, respectively.

**Table 1:** Anthropometric characteristics of the study population, according to sex and school grade

	N	Age (years)	Weight (kg)	Height (cm)	**BMI (kg/m**^**2**^**)**	Overweight including obesity (%)*
**Boys**						
Kindergarten	3,980	5.5 (0.4)	19.4 (3.5)	112.8 (5.7)	15.2 (2.0)	9.7 (8.8 - 10.6)
4^th ^grade	4,489	9.2 ( 0.4)	29.5 (7.0)	133.8 (6.3)	16.3 (3.0)	12.4 (11.5 - 13.4)
7^th ^grade	4,262	12.5 (0.4)	42.2 (11.0)	151.8 (8.4)	18.2 (3.7)	13.2 (12.2 - 14.2)
10^th ^grade	3,941	15.7 (0.4)	57.7 (11.3)	169.7 (7.6)	20.0 (3.3)	10.0 (9.0 - 10.7)
**Girls**						
Kindergarten	3,735	5.5 (0.4)	19.0 (3.3)	111.9 (5.8)	15.1 (2.0)	12.3 (11.2 - 13.3)
4^th ^grade	4,411	9.2 (0.4)	30.1 (7.8)	133.8 (6.8)	16.7 (3.4)	17.8 (16.6 - 18.8)
7^th ^grade	4,276	12.5 (0.4)	46.0 (11.5)	154.0 (7.2)	19.2 (4.1)	19.0 (17.8 - 20.2)
10^th ^grade	4,246	15.7 (0.4)	54.9 (11.9)	160.2 (6.4)	21.3 (4.2)	18.1 (17.0 - 19.3)

*Overweight and obesity are defined according to the IOTF criteria [9]

Results are expressed as mean (SD) for continuous variables and as percentage (95% confidence interval) for prevalence.

**Figure 1:**
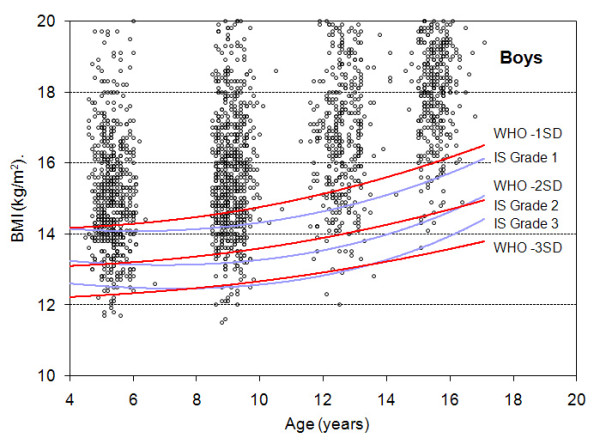
**Distribution of BMI-for-age in boys and comparison of the IS and WHO cut-offs**. The Y axis is truncated at BMI 20 kg/m^2^.

**Figure 2:**
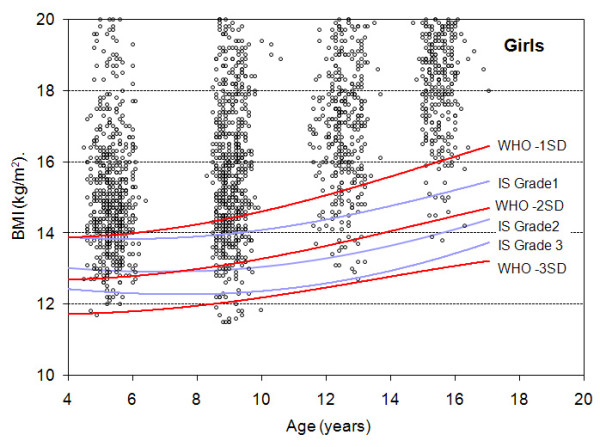
**Distribution of BMI-for-age in girls and comparison of the IS and WHO cut-offs**. The Y axis is truncated at BMI 20 kg/m^2^

Figures 1 and 2 display, in boys and girls respectively, the scatter plot distribution of BMI according to age and the curves corresponding to the cut-offs for the three thinness categories for both the IS and the WHO growth references. In order to distinguish individual observations, data are restricted to the year 2004 (2,446 boys and 2,408 girls).

Table 2 shows the prevalence of thinness categories in boys and girls, according to the IS and the WHO cut-offs in the whole sample. Overall (boys and girls together), the prevalence of the first thinness category was higher with the WHO than with the IS cut-offs (25.7% vs. 21.4%), no difference was observed for the second thinness category with either IS or WHO cut-offs (6.7% vs. 6.4%) and the prevalence of the third thinness category was lower with the WHO cut-offs than with the IS cut-offs (1.2% vs. 2.0%). The prevalence of the second and third thinness categories was higher in girls with the IS than with the WHO cut-offs, whereas the prevalence for the second thinness category was higher in boys with the WHO cut-offs than with the IS cut-offs. Of note, sex differences in the prevalence of the second and third thinness categories were small in absolute terms.

**Table 2:** Prevalence of thinness categories based on the IS cut-offs and the WHO cut-offs

Categories		Boys (n = 16,672)	Girls (n = 16,668)	Total (n = 33,340)
**IS cut-offs**				
Grade 1	<18.5 kg/m^2 ^*	22.5 (21.9 - 23.2)	20.3 (19.7 - 20.9)	21.4 (21.0 - 21.9)
Grade 2	<17.0 kg/m^2 ^*	6.2 (5.8 - 6.6)	6.6 (6.2 - 7.0)	6.4 (6.2 - 6.7)
Grade 3	<16.0 kg/m^2 ^*	1.6 (1.4 - 1.8)	2.3 (2.1 - 2.6)	2.0 (1.8 - 2.1)
**WHO cut-offs**				
	<-1 SD	27.8 (27.1 - 28.5)	23.7 (23.0 - 24.3)	25.7 (25.2 - 26.2)
Thinness	<-2 SD	7.7 (7.3 - 8.1)	5.7 (5.4 - 6.1)	6.7 (6.4 - 7.0)
Severe thinness	<-3 SD	1.4 (1.2 - 1.6)	1.0 (0.9 - 1.2)	1.2 (1.1 - 1.3)

IS cut-offs: criteria from an international survey [7]; WHO cut-offs: criteria from the World Health Organization [8]; SD: standard deviation.

Results are expressed as percentage (95% confidence interval).

*at age 18 years

**Figure 3:**
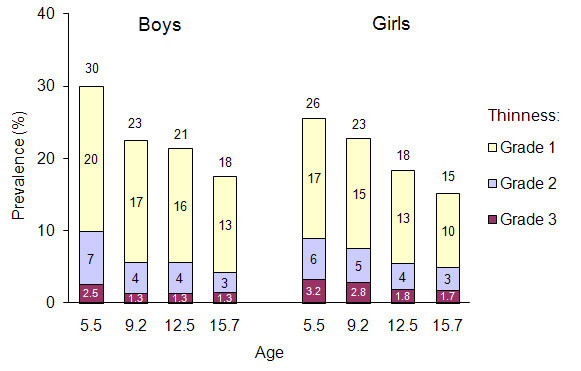
**Prevalence of grades of thinness according to age and gender according to the IS cut-offs**.

**Figure 4:**
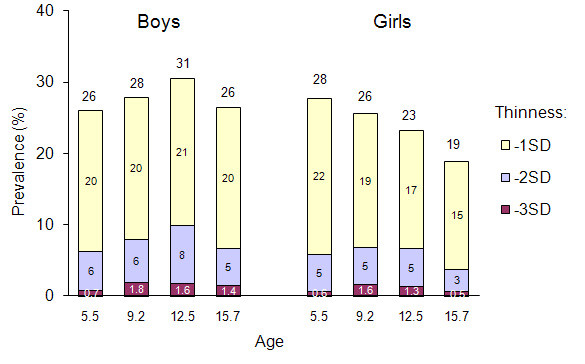
**Prevalence of grades of thinness according to age and gender according to the WHO cut-offs**.

The prevalence of categories of thinness by age and gender is summarized in Figure 3 (IS cut-offs) and in Figure 4 (WHO cut-offs). Several observations can be made when comparing the two figures. First, there was no large overall difference when comparing the IS and the WHO classifications: the prevalence of the first thinness category, by age and sex, ranged between 15-30%, the prevalence of the second thinness category ranged between 3-10% and the prevalence of the third category ranged between 0.5-3%. Second, the prevalence of the first thinness category was higher with the WHO than with the IS cut-offs for most age and sex categories while there was no marked difference between either cut-offs with the two most stringent categories. Third, the prevalence of all thinness categories tended to decrease according to age, particularly with the IS cut-offs.

## Discussion

Two growth references enabling the assessment of three thinness categories in school-going children have been issued recently based on different samples and different ways to assess the cut-offs [7,8]. The IS categories refer to thinness of grades 1, 2 and 3 (which reflect on how current BMI would correspond to BMI at age 18) while the WHO growth reference allows to define three thinness categories (based on BMI for age set at -1, -2, and -3 SD) with the second and third categories referred to as "thinness" and "severe thinness". Among children in the Seychelles aged 5 to 16 years, the prevalence of the first thinness category was higher with the WHO than with the IS cut-offs (25.7% vs. 21.4%), while no marked difference, in absolute terms, was observed when comparing the second (6.7% vs. 6.4%) and third (1.2% vs. 2.0%) thinness categories according to either classification. The prevalence of thinness categories tended to change with age for both sexes for the IS reference and among girls for the WHO reference. This first comparison of the IS and WHO cut-offs of thinness in a population-based sample of children in a country in the African region extends the yet limited knowledge in other regions [13-16].

Because they provide a common denominator, both the IS and WHO thinness categories allow to compare nutritional status across countries, follow trends, and look at global patterns [24]. It must be remembered that normative references are not meant for value judgement and, accordingly, the interpretation related to health significance is not the main objective of the IS or WHO cut-offs. While elevated BMI in youth is clearly linked with a variety of morbidity outcomes [25], the clinical significance of thinness categories in children and adolescents is still scarce [26-29]. In a previous study in Seychelles, we showed substantially lower physical fitness among both overweight and thin children, as compared to "normal" weight children [5].

Determining the clinical value of thinness categories is complicated by several factors i.e. changes in body composition with growth [30,31], variation in body frame size, relative leg length or muscle mass across populations [32,33] and differences in timing and tempo of adolescent growth spurt, peak height velocity and sexual maturation across populations [34].

In the absence of a gold standard, the development of thinness categories is challenging. Universal growth cut-offs (both thinness and overweight) should be based on data collected in populations that don't suffer from either excessive overweight or underweight problems [24,35,36]. The "true" prevalence of a specified thinness indicator (e.g. "severe thinness") would be underestimated if the base population from which the indicator is derived was undernourished and, inversely, overestimated if there was no under-nutrition problem in that reference population. The WHO cut-offs take these factors into consideration since growth references are based on data collected both before the obesity epidemic and among non-deprived children. This also applies, partly, to the IS cut-offs that are based on data mainly collected before the upswing of the obesity epidemic. Building new standards or updating old growth curves would need appropriate adjustment to correct for the secular upward shift in population mean BMI along the overweight epidemic worldwide [29].

The strengths of this study include a large sample size, a population-based design, a high participation rate and a broad range in the age of children and adolescents. A limitation is that weight and height were measured within a routine surveillance program. This may incur some random measurement error that may have impact on the estimates when studying the distribution of extreme values (e.g. severe thinness). Further studies should examine whether categories of thinness are related to ethnic differences (and if yes in what way), when using the BMI as an indicator.

## Conclusion

In this population of children and adolescents aged 5-16 in the Seychelles, the prevalence of thinness based on a deviation of one standard deviation of BMI for age (WHO) was higher than the prevalence of thinness of grade 1 with the IS cut-offs. The prevalence of thinness of grades 2 and 3, assessed with the IS cut-offs, was not largely different than the prevalence of "thinness" and "severe thinness", assessed with the WHO cut-offs. The prevalence of thinness tended to change according to children's age and there were also some differences according to sex. Similar studies should be conducted in other populations in the region. More generally, findings in diverse populations should be reviewed in order to provide general guidance for health professionals on the use of these thinness standards. Furthermore, while universal cut-offs derived from normative approaches are useful for international comparison, studies should clarify the health significance of the different thinness categories.

### What is known

•	Two universal references for assessing thinness in children and adolescents have been recently published (referred as the "WHO" cut-offs and the "IS" cut-offs), based on different normative (statistical) procedures, and each definition provides three categories of thinness

•	With a common denominator, both the IS and WHO thinness categories allow to compare nutritional status across countries, follow trends and look at global patterns

•	Few population-based studies have compared the IS and WHO cut-offs and none in the African region

### What the study adds

•	The prevalence of the first thinness category was higher with the WHO cut-offs than with the IS cut-offs while there was no marked difference, in absolute terms, between the second and third thinness categories with either cut-offs

•	The prevalence of thinness categories decreased along increasing child's age for both sexes using the IS reference and for girls using the WHO references

•	Further studies are needed to assess the health significance of these thinness categories in different populations

## Competing interests

The authors declare that they have no competing interests.

The views expressed by the authors of this paper do not reflect necessarily those of their institutions.

## Authors' contributions

PB designed the study, analyzed the data and co-wrote the manuscript; NK co-wrote the manuscript; GM organized the data collection; MB and AC critically reviewed the manuscript. All authors read and approved the final manuscript.

## References

[B1] Ezzati M Lopez AD Rodgers A Vander Hoorn S Murray CJ Selected major risk factors and global and regional burden of disease Lancet 2002 360 1347 1360 10.1016/S0140-6736(02)11403-6 12423980

[B2] Pelletier DL The potentiating effects of malnutrition on child mortality: epidemiologic evidence and policy implications Nutr Rev 1994 52 409 415 7898782 10.1111/j.1753-4887.1994.tb01376.x

[B3] Misra M Aggarwal A Miller KK Almazan C Worley M Soyka LA Herzog DB Klibanski A Effects of anorexia nervosa on clinical, hematologic, biochemical, and bone density parameters in community-dwelling adolescent girls Pediatrics 2004 114 1574 1583 10.1542/peds.2004-0540 15574617

[B4] Luder E Alton I Strang J, Story M The underweight adolescent Guidelines for Adolescent Nutritional Services 2005 Minnesota: University of Minnesota 93 100

[B5] Bovet P Auguste R Burdette H Strong inverse association between physical fitness and overweight in adolescents: a large school-based survey Int J Behav Nutr Phys Act 2007 4 24 10.1186/1479-5868-4-24 17550617 PMC1894813

[B6] Ferrar K Olds T Thin adolescents: Who are they? What do they do? Socio-demographic and use-of-time characteristics Prev Med 2010 51 253 258 10.1016/j.ypmed.2010.07.001 20630482

[B7] Cole TJ Flegal KM Nicholls D Jackson AA Body mass index cut offs to define thinness in children and adolescents: international survey BMJ 2007 335 194 10.1136/bmj.39238.399444.55 17591624 PMC1934447

[B8] de Onis M Onyango AW Borghi E Siyam A Nishida C Siekmann J Development of a WHO growth reference for school-aged children and adolescents Bull World Health Organ 2007 85 660 667 10.2471/BLT.07.043497 18026621 PMC2636412

[B9] Cole TJ Bellizzi MC Flegal KM Dietz WH Establishing a standard definition for child overweight and obesity worldwide: international survey BMJ 2000 320 1240 1243 10.1136/bmj.320.7244.1240 10797032 PMC27365

[B10] Hamill PV Drizd TA Johnson CL Reed RB Roche AF NCHS growth curves for children birth-18 years. United States Vital Health Stat 1977 11 i iv 1-74 611680

[B11] WHO Multicentre Growth Reference Study Group WHO Child Growth Standards: length/height-for-age, weight-for-age, weight-for length, weight-for-height and body mass index-for-age: methods and development 2006 Geveva: WHO

[B12] WHO Multicentre Growth Reference Study Group WHO Child Growth Standards based on length/height, weight and age Acta Paediatr Suppl 2006 450 76 85 16817681 10.1111/j.1651-2227.2006.tb02378.x

[B13] Gomes Fda S Anjos LA Vasconcellos MT Influence of different body mass index cut-off values in assessing the nutritional status of adolescents in a household survey Cad Saude Publica 2009 25 1850 1857 19649426 10.1590/s0102-311x2009000800021

[B14] Silva HG Chiara VL Barros ME Rego AL Ferreira A Pitasi BA Mattos T Diagnosing the nutritional status of schoolchildren: a comparison between Brazilian and international criteria J Pediatr (Rio J) 2008 84 550 555 10.2223/JPED.1853 19060985

[B15] Baya Botti A Perez-Cueto FJ Vasquez Monllor PA Kolsteren PW International BMI-for-age references underestimate thinness and overestimate overweight and obesity in Bolivian adolescents Nutr Hosp 2010 25 428 436 20593126

[B16] Khasnutdinova SL Grjibovski AM Prevalence of stunting, underweight, overweight and obesity in adolescents in Velsk district, north-west Russia: a cross-sectional study using both international and Russian growth references Public Health 2010 124 392 397 10.1016/j.puhe.2010.03.017 20541233

[B17] Best C Neufingerl N van Geel L van den Briel T Osendarp S The nutritional status of school-aged children: why should we care? Food and nutrition bulletin 2010 31 400 417 20973461 10.1177/156482651003100303

[B18] Bovet P Shamlaye C Gabriel A Riesen W Paccaud F Prevalence of cardiovascular risk factors in a middle-income country and estimated cost of a treatment strategy BMC Public Health 2006 6 9 10.1186/1471-2458-6-9 16423280 PMC1379635

[B19] Bovet P Chiolero A Madeleine G Gabriel A Stettler N Marked increase in the prevalence of obesity in children of the Seychelles, a rapidly developing country, between 1998 and 2004 Int J Pediatr Obes 2006 1 120 128 10.1080/17477160600761068 17907325

[B20] Marques-Vidal P Madeleine G Romain S Gabriel A Bovet P Secular trends in height and weight among children and adolescents of the Seychelles, 1956-2006 BMC Public Health 2008 8 166 10.1186/1471-2458-8-166 18489755 PMC2405790

[B21] Stettler N Bovet P Shamlaye H Zemel BS Stallings VA Paccaud F Prevalence and risk factors for overweight and obesity in children from Seychelles, a country in rapid transition: the importance of early growth Int J Obes Relat Metab Disord 2002 26 214 219 10.1038/sj.ijo.0801860 11850753

[B22] Bovet P Chiolero A Shamlaye C Paccaud F Prevalence of overweight in the Seychelles: 15 year trends and association with socio-economic status Obes Rev 2008 9 511 517 10.1111/j.1467-789X.2008.00513.x 18673305

[B23] Chiolero A Paradis G Madeleine G Hanley JA Paccaud F Bovet P Discordant secular trends in elevated blood pressure and obesity in children and adolescents in a rapidly developing country Circulation 2009 119 558 565 10.1161/CIRCULATIONAHA.108.796276 19153270

[B24] de Onis M The use of anthropometry in the prevention of childhood overweight and obesity Int J Obes Relat Metab Disord 2004 28 Suppl 3 S81 85 15543225 10.1038/sj.ijo.0802810

[B25] Han JC Lawlor DA Kimm SY Childhood obesity Lancet 2010 375 1737 1748 10.1016/S0140-6736(10)60171-7 20451244 PMC3073855

[B26] Mei Z Grummer-Strawn LM Pietrobelli A Goulding A Goran MI Dietz WH Validity of body mass index compared with other body-composition screening indexes for the assessment of body fatness in children and adolescents Am J Clin Nutr 2002 75 978 985 12036802 10.1093/ajcn/75.6.978

[B27] Duggan MB Anthropometry as a tool for measuring malnutrition: impact of the new WHO growth standards and reference Ann Trop Paediatr 2010 30 1 17 10.1179/146532810X12637745451834 20196929

[B28] Freedman DS Wang J Maynard LM Thornton JC Mei Z Pierson RN Dietz WH Horlick M Relation of BMI to fat and fat-free mass among children and adolescents Int J Obes (Lond) 2005 29 1 8 10.1038/sj.ijo.0802735 15278104

[B29] Lobstein T Baur L Uauy R Obesity in children and young people: a crisis in public health Obes Rev 2004 5 Suppl 1 4 104 15096099 10.1111/j.1467-789X.2004.00133.x

[B30] Rosario AS Kurth BM Stolzenberg H Ellert U Neuhauser H Body mass index percentiles for children and adolescents in Germany based on a nationally representative sample (KiGGS 2003-2006) Eur J Clin Nutr 2010 64 341 349 10.1038/ejcn.2010.8 20179728

[B31] Wang Y Wang JQ A comparison of international references for the assessment of child and adolescent overweight and obesity in different populations Eur J Clin Nutr 2002 56 973 982 10.1038/sj.ejcn.1601415 12373618

[B32] Deurenberg P Deurenberg-Yap M Guricci S Asians are different from Caucasians and from each other in their body mass index/body fat per cent relationship Obes Rev 2002 3 141 146 10.1046/j.1467-789X.2002.00065.x 12164465

[B33] Deurenberg P Universal cut-off BMI points for obesity are not appropriate Br J Nutr 2001 85 135 136 10.1079/BJN2000273 11280336

[B34] Malina RM Katzmarzyk PT Validity of the body mass index as an indicator of the risk and presence of overweight in adolescents Am J Clin Nutr 1999 70 131S 136S 10393160 10.1093/ajcn/70.1.131s

[B35] Bovet P Chiolero A Madeleine G Paccaud F Prevalence of overweight and underweight in public and private schools in the Seychelles Int J Pediatr Obes 2010 5 274 278 10.3109/17477160903449986 20184505

[B36] Butte NF Garza C de Onis M Evaluation of the feasibility of international growth standards for school-aged children and adolescents J Nutr 2007 137 153 157 17182818 10.1093/jn/137.1.153

